# Interaction of Caveolin-1 with Ku70 Inhibits Bax-Mediated Apoptosis

**DOI:** 10.1371/journal.pone.0039379

**Published:** 2012-06-20

**Authors:** Huafei Zou, Daniela Volonte, Ferruccio Galbiati

**Affiliations:** Department of Pharmacology and Chemical Biology, University of Pittsburgh School of Medicine, Pittsburgh, Pennsylvania, United States of America; Sun Yat-sen University Cancer Center, China

## Abstract

Caveolin-1, the structural protein component of caveolae, acts as a scaffolding protein that functionally regulates signaling molecules. We show that knockdown of caveolin-1 protein expression enhances chemotherapeutic drug-induced apoptosis and inhibits long-term survival of colon cancer cells. *In vitro* studies demonstrate that caveolin-1 is a novel Ku70-binding protein, as shown by the binding of the scaffolding domain of caveolin-1 (amino acids 82–101) to the caveolin-binding domain (CBD) of Ku70 (amino acids 471–478). Cell culture data show that caveolin-1 binds Ku70 after treatment with chemotherapeutic drugs. Mechanistically, we found that binding of caveolin-1 to Ku70 inhibits the chemotherapeutic drug-induced release of Bax from Ku70, activation of Bax, translocation of Bax to mitochondria and apoptosis. Potentiation of apoptosis by knockdown of caveolin-1 protein expression is greatly reduced in the absence of Bax expression. Finally, we found that overexpression of wild type Ku70, but not a mutant form of Ku70 that cannot bind to caveolin-1 (Ku70 Φ→A), limits the chemotherapeutic drug-induced Ku70/Bax dissociation and apoptosis. Thus, caveolin-1 acts as an anti-apoptotic protein in colon cancer cells by binding to Ku70 and inhibiting Bax-dependent cell death.

## Introduction

Caveolae are 50 to 100 nm flask-shaped invaginations of the plasma membrane enriched in cholesterol and glycosphingolipids. Caveolae can exist as individual invaginations or are found in detached grape-like clusters and long tubular structures derived from the fusion of single caveolae within the cytoplasm. Caveolae were identified in the 1950’s in capillary endothelial cells and epithelial cells from the gall bladder of the mouse [1], [2]. Since then, caveolae have been found in a wide variety of tissues and cell types, including adipocytes, endothelial cells, striated and smooth muscle cells, fibroblasts and epithelial cells [3]. Caveolae were originally thought to function as macromolecular transport vesicles [4]. From then on, their role has expanded to signal transduction, cellular metabolism, cholesterol homeostasis, endocytosis and tumor suppression [5]–[9].

Caveolin-1 is an essential protein component of caveolae. Caveolin-1 acts as a scaffolding protein that concentrates and functionally regulates signaling molecules, including G-protein alpha subunits, H-Ras, Nitric Oxide Synthase (NOS), Epidermal Growth Factor Receptor (EGFR), Src-like Nonreceptor Tyrosine Kinases (NRTK), Protein Kinase C (PKC), Protein Kinase A (PKA), Mdm2, Thioredoxin Reductase 1 and Protein Phosphatase type 2A [10]–[13]. The direct interaction between caveolin-1 and signalling molecules occurs between the “caveolin scaffolding domain" (CSD) of caveolin-1, which is represented by residues 82–101, and the caveolin binding domain (CBD: ΦXΦXXXXΦ, ΦXXXXΦXXΦ, or ΦXΦXXXXΦXXΦ where Φ represents an aromatic amino acid and X represents any amino acid) of signaling molecules [14], [15]. The direct interaction with caveolin-1 generally results in the sequestration of a given signaling molecule within caveolar membranes and inhibition of its signaling activity.

Colorectal cancer is the fourth most commonly diagnosed cancer and the second leading cause of cancer-related deaths in the United States [16], [17]. Apoptosis is an anti-tumorigenic event. In fact, tumor cells need to escape apoptosis in order to generate a relevant tumor mass. Thus, apoptosis has a profound effect on the malignant phenotype. In addition, most cytotoxic anticancer agents promote apoptosis, suggesting that defective apoptotic programs may contribute to treatment failure, which is commonly seen in most human cancers. Studies have shown that caveolin-1 is over-expressed in colon cancer tissues, as compared to normal colonic mucosa, and colon cancer cells [18]–[20], suggesting that caveolin-1 may have pro-tumorigenic properties in this type of cancer. However, whether and how caveolin-1 modulates apoptosis of colon cancer cells remains to be determined.

An important regulatory step in apoptosis is the activation of the proapoptotic factor Bax, which translocates to the outer mitochondrial membrane and oligomerizes following its activation. Bax promotes apoptosis by making the membrane permeable to cytochrome c. Ku70 is a protein that was originally identified as part of the Ku70/Ku80 complex that mediates the repair of DNA double-strand breaks by non-homologous end joining (NHEJ) [21]. However, Ku70 has also been shown to regulate apoptosis through a Bax-dependent mechanism. Over-expression of Ku70 inhibits Bax-mediated apoptosis, whereas depletion of Ku70 enhances apoptosis [22]. In addition, Ku70 −/− mice are hypersensitive to ionizing radiation [23] and cells from these mice are also hypersensitive to apoptosis induced by staurosporine [24]. Increased acetylation of Ku70 disrupts the Ku70-Bax interaction and inhibits the antiapoptotic function of Ku70 [25]. Finally, peptides based on the amino acid sequence of the Bax-binding domain of Ku70 bind Bax and inhibit cell death [26], [27]. Thus, by interacting with Ku70, Bax is sequestered away from mitochondria and is maintained in an inactive state. The molecular mechanism underlying the sequestration of Bax by Ku70 and the release of Bax from Ku70 after stimulation with an apoptotic inducer remains to be fully identified.

In this study, we focused on the functional role of caveolin-1 in chemotherapeutic-induced apoptosis of colon cancer cells and the underlying molecular mechanism. We found that Ku70 is a novel caveolin-1-binding protein and that binding of caveolin-1 to Ku70 inhibits chemotherapeutic drug-induced apoptosis of colon cancer cells by limiting the release of Bax from Ku70.

## Materials and Methods

### Materials

Antibodies and their sources were as follows: anti-caveolin-1 (2297) and anti-Bax (6A7) IgGs were from BD Transduction Laboratories (Lexington, KY); anti-caveolin-1 (H-97), anti-Ku70 (A-9) and anti-Bax (N-20) IgGs were from Santa Cruz Biotechnology (Santa Cruz, CA); anti-cleaved caspase-3 and anti-cleaved PARP IgGs were from Cell Signaling Technology (Danvers, MA); horseradish peroxidase-conjugated goat anti-mouse and anti-rabbit secondary antibodies were from Pierce (Rockford, IL, USA). McCoy’s 5a medium and fetal bovine serum were purchased from Invitrogen (Carlsbad, CA). Other biochemicals used were of the highest purity available and were obtained from regular commercial sources.

### Cell Culture and Drug Treatment

Human colon carcinoma cells HT29, HCT116 Bax +/− (HCT116) and HCT116 Bax −/− (HCT116 Bax null) (a generous gift from Dr. Bert Vogelstein) were grown in McCoy’s 5a medium containing glutamine, antibiotics and 10% FBS. Cells were incubated in growth medium supplemented with 50, 25, 12.5, 6.25, or 3.125 µg/ml of 5-FU for 12, 24 or 48 hours. HCT116 and HCT116 Bax null cells were also incubated in growth medium supplemented with 800, 600, 400 or 200 µM of etoposide for 24 hours.

### siRNA Treatment

Knockdown of caveolin-1 protein expression was achieved by transfection of HCT116 and HT29 cells with small interfering RNA (siRNA) duplexes. The target sequence was as follows: 5′-aaccagaagggacacacag-3′. Scrambled siRNA was used as a negative control. Transfection was performed using Lipofectamine 2000 according to the manufacture’s recommendations.

#### Cloning of wild type (WT) and Φ→A Ku70 constructs

The entire coding region of mouse Ku70 was amplified by polymerase chain reaction amplification using a full-length cDNA clone (ID 3669715) from Open Biosystems (Huntsville, AL) and the following primers: forward 5′-gaattcggtcagagtgggagtcctactac-3′ and reverse 5′-ctcgagtcagttcttctccaagtgtct-3′. The amplified DNA was sequenced to rule out the introduction of unwanted mutations. The PCR fragment was digested to create unique EcoRI and XhoI ends and subcloned into the pCMV-HA expression vector (WT Ku70-HA). The Φ→A mutant of Ku70 (Φ→A Ku70-HA) was generated by replacement of the aromatic residues within the caveolin binding motif of Ku70 (amino acids 471–478) with alanines by polymerase chain reaction amplification using appropriate internal primers (forward: 5′-gttcaaaagctccgcgccacagccagatctgacagtgctgagaatccagtcctg-3′; reverse: 5′-caggactggattctcagcactgtcagatctggctgtggcgcggagcttttgaac-3′) and subcloned EcoRI and XhoI into the pCMV-HA expression vector. Transfection of WT Ku70-HA and Φ→A Ku70-HA in HCT116 cells was performed using Lipofectamine 2000 according to the manufacture’s recommendations.

### Western Blotting Assay

Protein samples were collected in boiling Laemmli buffer (0.2 M Tris-HCl, pH 6.8, 2% SDS, 10% glycerol), vigorously resuspended and boiled for 5 min. Protein concentration was calculated using the BCA protein assay kit (Thermo Fisher Scientific Inc., Rockford, IL). Equal amount of proteins were separated on 12.5% Tris-HCl polyacrylamide gels after the addition of 0.01% bromophenol blue and 20 mM DTT. Protein samples were then transferred onto nitrocellulose membranes overnight at 4°C. Ponceau S staining was performed to confirm equal loading. Blots were incubated for 1 hr in TBST (10 mM Tris-HCl, pH 8.0, 150 mM NaCl, and 0.2% Tween 20) containing 2% dry milk and 1% bovine serum albumin. After three washes with TBST, membranes were incubated overnight with the primary antibody and for 1 hr with horseradish peroxidase-conjugated secondary IgG. Bound antibodies were detected using a Super Signal chemiluminescent solution (Pierce Chemical, Rockford, IL).

### DAPI (4,6-Diamidino-2-Phenylindole) Staining

HCT116 and HT29 cells (untreated or treated with chemotherapeutic drugs) were harvested and incubated with staining buffer (PBS containing 3.7% paraformaldehyde, 0.1% Triton, 10 µg/ml RNase A and 1 µg/ml DAPI) at room temperature for 1 hr. Nuclear morphology was examined under a Olympus Provis fluorescent microscope. A total of 1,200 cells were scored from 4 independent viewing areas from three independent experiments for each experimental point.

#### Annexin V staining and flow cytometry analysis

HCT116 cells were transfected with caveolin-1 siRNA and scrambled siRNA as described above. After 24 hours, cells were treated with 25 µg/ml 5-FU for 48 hours. Untreated cells were used as control. The Annexin-V-FITC Apoptosis Detection Kit (BD Pharmingen, San Diego, CA) was used to detect apoptosis by flow cytometry. Cells were harvested (including detached cells), and processed according to the manufacturer’s instructions. Fluorescence-activated cell sorting analysis was carried out using a FACScan flow cytometer (Becton Dickinson, San Diego, CA) and CellQuest software.

### Clonogenic Assay

HCT116 cells were transfected with either caveolin-1 siRNA or scrambled siRNA as described above. After 24 hours, cells were treated with different concentrations of either 5-FU (3.125 µg/ml, 6.25 µg/ml, 12.5 µg/ml and 25 µg/ml) or etoposide (200 µM, 400 µM and 600 µM) for 1 hour. Untreated cells were used as control. 500 cells were then plated into 12-well plates and cultured for 7 days. Cells were stained with crystal violet by incubating the cells with 10% crystal violet in 70% ethanol for 2 minutes followed by extensive washes with PBS. Quantification of crystal violet staining from three independent experiments was performed by counting the number of clones using Image J software analysis.

### Cell Cycle Analysis by Fluorescence-activated Cell Sorter (FACS)

Cells were collected by trypsinization, pelleted, and washed with PBS. Cells were resuspended and fixed overnight with 1 ml of ice-cold 70% ethanol at 4°C. On the day of analysis, cells were centrifuged at 3000 rpm for 8 min and the supernatant was carefully removed. Subsequently, cells were resuspended in 1.0 ml of PI solution (PBS containing 0.1% glucose, 1.1 mg/ml RNAse, and 0.05 mg/ml propidium iodide) and incubated at room temperature for 30 min. Cell were then subjected to FACS analysis with the use of a flow cytometer (LSR II, BD biosciences). For each analysis 10,000 gated events were collected to permit cell cycle analysis. Data analysis was performed with the use of CellQuest software. A representative cell cycle analysis from three independent experiments is shown.

### Glutathione ***S***-transferase (GST) Pull-down Assay

GST-caveolin-1 (GST-Cav-1) constructs were as follows: GST fused to full-length caveolin-1 (Cav-1 FL), GST fused to residues 82 to 101 of caveolin-1 (GST-Cav-1 (82–101)) and GST fused to residues 1 to 101 of caveolin-1 (GST-Cav-1 (1–101)). GST-Cav-1 fusion protein constructs were transformed into *Escherichia coli* (BL21 strain; Novagen, Inc.). After induction of expression through addition of 0.5 mM isopropyl-β-D-galactoside (Sigma), GST-Cav-1 proteins were affinity purified on glutathione-agarose beads, using the detergent Sarcosyl for initial solubilization. GST-Cav-1 and GST alone (bound to glutathione-agarose beads) were washed 3 times with TNET buffer (50 mM Tris, pH 8.0, 150 mM NaCl, 5 mM EDTA, 1% Triton X-100) containing protease inhibitors. SDS-PAGE followed by Comassie staining was used to determine the concentration of GST-Cav-1 per 100 µl of packed bead volume. Pre-cleared cell lysates of HCT116 cells over-expressing HA-Ku70 were diluted in buffer A (10 mM Tris, pH 8.0, 0.1% Tween 20) and added to approximately 100 µl of equalized bead volume for overnight incubation at 4°C. After binding, the beads were extensively washed with phosphate-buffered saline (6 times). Finally, the beads were resuspended in 2X sample buffer (0.4 M Tris-HCl, pH 6.8, 4% SDS, 20% glycerol, 0.02% bromophenol blue) and subjected to SDS-PAGE.

### Co-Immunoprecipitation

Cells were washed twice with PBS and lysed for 45 min at 4°C in an IP lysis buffer (10 mM Tris, pH 8.0, 150 mM NaCl, 5 mM EDTA, 1% Triton X-100, 60 mM octyl glucoside and protease inhibitors; Bax immunoprecipitation was performed using 0.1 mM Hepes and 1% CHAPS). Subsequently, lysates were centrifuged at 13,000×g for 10 min at 4°C. The supernatant was pre-incubated with protein A sepharose beads for 1 hr at 4°C. After centrifugation at 10,000 rpm for 30 sec, the supernatant was collected and protein concentration calculated using the BCA protein assay kit (Thermo Fisher Scientific Inc.). Equal amount of total proteins were incubated overnight at 4°C with anti-caveolin-1 or anti-Bax antibodies and protein A sepharose beads. The immunoprecipitates were washed three times with lysis buffer and re-suspended in Laemmli buffer (0.2 M Tris-HCl, pH 6.8, 2% SDS, 10% glycerol, 0.01% bromophenol blue, 20 mM DTT). Following vigorous vortexing and boiling, samples were separated on 12.5% SDS-PAGE. Blots were then probed with appropriated antibodies. Immunoprecipitations with protein A sepharose beads were performed as control and did not produce any detectable band by immunoblotting analysis using the appropriate antibody (data not shown).

### Mitochondria Isolation

Cells were washed once in PBS and resuspended in 600 µL of mitochondrial isolation buffer containing 10 mM Hepes (pH 7.4), 250 mM sucrose, 1 mM EDTA and protease inhibitors. Cells were broken in a Dounce homogenizer for 40 times. Nuclei and unbroken cells were removed by centrifugation at 1000×g at 4°C for 15 min. Clarified lysates were subjected to centrifugation at 12,000×g at 4°C for 15 min. The mitochondrion-containing pellets were resuspended in 2x SDS sample buffer. Mitochondrial fractions were positive for the mitochondria marker cytochrome oxidase subunit IV and negative for the cytoplasmic marker alpha-tubulin (data not shown).

### RNA Isolation and RT-PCR

Cells were collected and total RNA was isolated using the RNeasy Mini kit from Qiagen (Valencia, CA). Equal amounts of RNA were treated with RNase-free DNase, and subjected to reverse transcription using the Advantage RT-for-PCR kit from Clontech (Mountain View, CA), according to the manufacturer’s recommendations. PCR was then performed in the exponential linear zone of amplification for each gene studied. The caveolin-1-specific primers used were the following: forward 5′-ctacaagcccaacaacaagg-3′; reverse 5′- cagacagcaagcggtaaaa-3′. A sequence corresponding to GAPDH was also amplified as an internal control.

### Immunofluorescence Microscopy

Cells grown on glass coverslips were washed three times with PBS w/Ca^++^/Mg^++^ and fixed for 30 min at room temperature with 2% paraformaldehyde in PBS w/Ca^++^/Mg^++^. Fixed cells were rinsed with PBS w/Ca^++^/Mg^++^and permeabilized with 0.1% Triton X-100, 0.2% bovine serum albumin for 10 min. Then cells were treated with 25 mM NH_4_Cl in PBS w/Ca^++^/Mg^++^ for 10 min at room temperature to quench free aldehyde groups. Cells were treated with RNase (200 µg/ml) for 10 minutes and with DAPI (1 µg/ml) for 20 minutes. Cells were rinsed with PBS w/Ca^++^/Mg^++^ and incubated with the primary antibody (diluted in PBS with 0.1% Triton X-100, 0.2% bovine serum albumin) for 2 h at room temperature. After three washes with PBS w/Ca^++^/Mg^++^ (10 min each), cells were incubated with the secondary antibody for 1 h at room temperature: lissamine rhodamine B sulfonyl chloride-conjugated goat anti-rabbit antibody (5 µg/ml) and fluorescein isothiocyanate-conjugated goat anti-mouse antibody (5 µg/ ml). Finally, cells were washed three times with PBS w/Ca^++^/Mg^++^ (10 min each wash) and slides were mounted with slow-Fade anti-fade reagent (Molecular Probes, Inc., Eugene, OR) and observed using a Zeiss Confocal Microscope (LSM 5 Pascal).

### Statistical Analysis

Quantification studies were performed in triplicate and the average ± standard error of the mean (S.E.M.) is shown. Significance was calculated using the Student’s t-test. Quantification of active Bax was obtained by scanning images of western blotting analysis from three independent experiments using Image J software. Immunoprecipitation and immunoblotting analysis were performed three independent times and representative blots are shown.

## Results

### Caveolin-1 Inhibits Chemotherapeutic Drug-induced Apoptosis and Promotes Long-term Survival of Colon Cancer Cells

**Figure 1:**
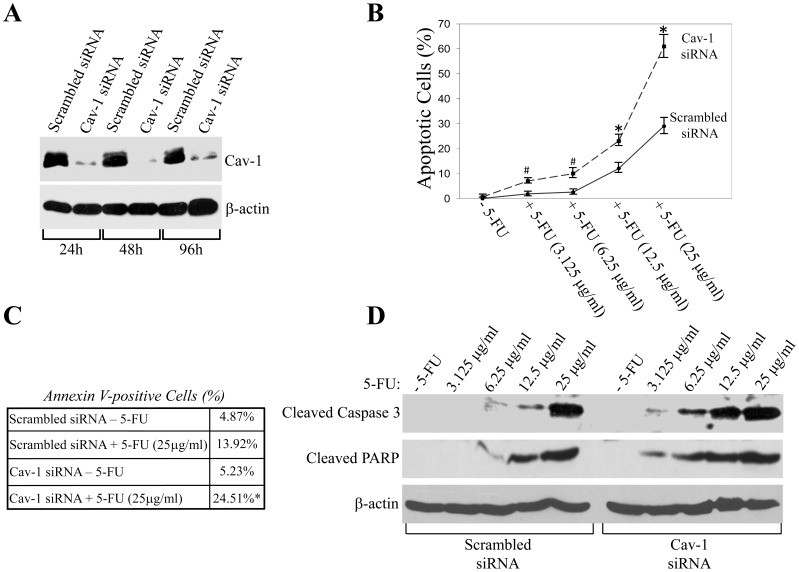
Knockdown of caveolin-1 protein expression sensitizes HCT116 colon cancer cells to apoptosis. HCT116 colon cancer cells were transfected with siRNA directed against caveolin-1. Transfection with scrambled siRNA was used as control. (**A**) Endogenous caveolin-1 expression was determined by immunoblotting analysis 24, 48 and 96 hours after transfection using an antibody probe specific for caveolin-1. Immunoblotting with anti-β-actin IgGs was done to show equal loading. (**B–D**) One day after transfection, HCT116 cells were treated with different concentrations of 5-FU for 48 hours. Untreated cells were used as control. In (**B**), cells were stained with DAPI and the number of cells showing nuclear condensation was quantified. Values represent mean ± SEM; *P<0.001; ^#^ *P*<0.005. In (**C**), cells were subjected to Annexin V staining and flow cytometry. Values represent mean. In (**D**), cells were subjected to immunoblotting analysis with antibody probes specific for cleaved caspase 3 and PARP. Immunoblotting with anti-β-actin IgGs was done to show equal loading.

To directly address the role of caveolin-1 in chemotherapeutic drug-induced apoptosis in colon cancer cells, we knocked-down caveolin-1 protein expression by siRNA in HCT116 colon cancer cells (Figure 1A). Scrambled and caveolin-1 siRNA-transfected HCT116 cells were treated with different concentrations (3.125 µg/ml, 6.25 µg/ml, 12.5 µg/ml and 25 µg/ml) of the chemotherapeutic drug 5-fluoracil (5-FU) for 48 hours (Figure 1B) and with the same concentration of 5-FU (25 µg/ml) for different periods of time (12 h, 24 h and 48 h) (Figures S1A and S1B). Cells were then stained with DAPI and apoptosis was quantified by counting the number of cells showing nuclear condensation. We show that 5-FU induced apoptosis in HCT116 cells transfected with scrambled siRNA and that 5-FU-induced apoptosis was significantly enhanced in HCT116 cells in which caveolin-1 protein expression was reduced by siRNA. These data were confirmed by annexin V staining followed by flow cytometry analysis (Figure 1C) and immunoblotting analysis using antibody probes specific for cleaved caspase 3 and Poly (ADP-ribose) polymerase (PARP) (Figure 1D). Studies have implicated reactive oxygen species (ROS) in 5-FU-mediated apoptosis in colon cancer cells [28]. To independently support our findings, we subjected HCT116 cells transfected with either scrambled or caveolin-1 siRNA to oxidative stress and apoptosis was quantified by DAPI staining. We found that knockdown of caveolin-1 protein expression potentiated oxidative stress-induced apoptosis in HCT116 cells (data not shown).

**Figure 2:**
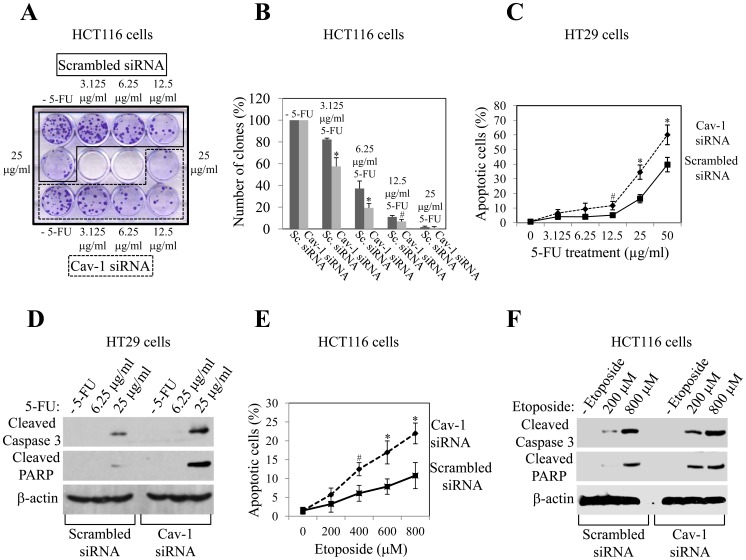
Caveolin-1 promotes long-term survival and inhibits apoptosis of colon cancer cells after treatment with chemotherapeutic drugs. HCT116 (A, B, E and F) and HT29 (C and D) colon cancer cells were transfected with siRNA directed against caveolin-1. Transfection with scrambled siRNA was used as control. One day after transfection, cells were treated with different concentrations of 5-FU for 48 h (A–D) or etoposide for 24 h (E and F). Untreated cells were used as control. In (A and B), cells were cultured for 7 days and stained with crystal violet. A crystal violet staining that is representative of three independent experiments is shown in (A), quantification of the number of clones after crystal violet staining is shown in (B). Values represent mean ± SEM; *P<0.001; ^#^ *P*<0.005. In (C and E), cells were stained with DAPI and the number of cells showing nuclear condensation was quantified. Values represent mean ± SEM; *P<0.001; ^#^ *P*<0.005. In (D and F), cells were subjected to immunoblotting analysis with antibody probes specific for cleaved caspase 3 and PARP. Immunoblotting with anti-β-actin IgGs was done to show equal loading.

Long-term clonogenic assays provide a more stringent measurement of cell survival than short-term assays. To determine the effect of reduced caveolin-1 expression on the long-term survival of colon cancer cells, HCT116 cells were transfected with either scrambled or caveolin-1 siRNA, treated with different concentrations of 5-FU (3.125 µg/ml, 6.25 µg/ml, 12.5 µg/ml and 25 µg/ml) and subjected to crystal violet staining 7 days after 5-FU treatment. Figures 2A and 2B show that knockdown of caveolin-1 protein expression reduced the long-term survival of HCT116 cells, even at the lowest concentration of 5-FU (3.125 µg/ml). Interestingly, the number of clones, after crystal violet staining, was reduced by the knockdown of caveolin-1 protein expression in HCT116 cells even before drug treatment (Figure 2A). Since caveolin-1 siRNA also reduced the number of cells in the S phase of the cell cycle by 13%, without promoting apoptosis, before treatment with 5-FU (Figure S2), caveolin-1 appears to have a positive effect on cell growth in HCT116 cells in addition to its anti-apoptotic function after stimulation with chemotherapeutic drugs. This is consistent with data showing pro-tumorigenic properties of caveolin-1 in colon cancer, as demonstrated by the over-expression of caveolin-1 in colon cancer tissues and colon cancer cells [18]–[20].

To determine whether the caveolin-1-mediated inhibition of apoptosis is not limited to the combination of HCT116 cells and 5-FU, a different colon cancer cell line, HT29, was treated with different concentrations of 5-FU after caveolin-1 protein expression was knockdown by siRNA (data not shown). Apoptosis was then quantified by DAPI staining (Figures 2C, S3A and S3B) and immunoblotting analysis to detect cleaved caspase 3 and PARP (Figure 2D). We found enhanced 5-FU-induced apoptosis when caveolin-1 protein expression was knockdown in HT29 cells. In addition, scrambled and caveolin-1 siRNA-transfected HCT116 cells were treated with a different chemotherapeutic drug, etoposide. We show enhanced etoposide-induced apoptosis in HCT116 cells transfected with caveolin-1 siRNA, as compared to scrambled siRNA-transfected cells, after DAPI staining (Figure 2E) and immunoblotting analysis to detect activated caspase 3 and PARP (Figure 2F). In addition, the long-term survival of HCT116 cells after treatment with etoposide was inhibited by knockdown of caveolin-1 protein expression using siRNA (Figures S4A and S4B).

### Ku70 is a Novel Caveolin-1-binding Protein

**Figure 3:**
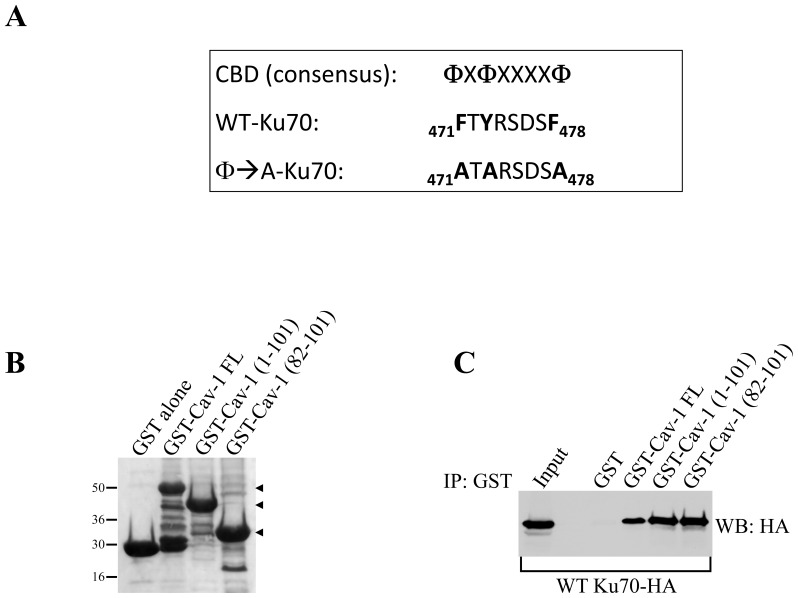
Ku70 binds to the scaffolding domain of caveolin-1. (**A**) The consensus caveolin binding domain (CBD) is shown together with the caveolin binding domain of Ku70 (amino acids 471 to 478) and a mutant form of Ku70’s CBD in which aromatic residues were mutated to alanines. (**B**) Ponceau S staining of GST alone and GST-caveolin-1 fusion proteins. (**C**) GST-caveolin-1 fusion protein pull-down assays were performed using cell lysates from HCT116 cells transiently transfected with wild type Ku70-HA. A blot that is representative of two independent experiments is shown.

Caveolin-1 is known to interact with signaling molecules carrying a caveolin binding domain [14], [15]. Ku70 has been shown to regulate apoptosis [22]–[25]. Interestingly, Ku70 possesses a putative caveolin binding domain between amino acids 471 and 478 (Figure 3A). To investigate the molecular mechanism underlying the caveolin-1-mediated inhibition of apoptosis, we asked whether Ku70 was a novel caveolin-1-interacting protein. To this end, we performed pulldown assays using a series of caveolin-1 deletion mutants fused to GST (Figure 3B). Figures 3C shows that Ku70 is a novel caveolin-1-binding protein and that the caveolin scaffolding domain (CSD) of caveolin-1 (residues 82–101) is sufficient for binding to Ku70.

### The Interaction between Caveolin-1 and Ku70 is Induced by Chemotherapeutic Drugs and Limits the Release of Bax from Ku70

**Figure 4:**
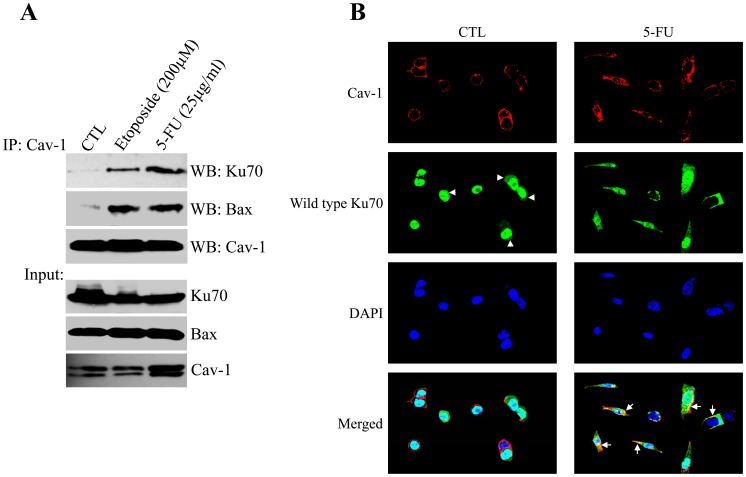
Chemotherapeutic drugs promote the Ku70-Cav-1 interaction. (**A**) HCT116 cells were treated with either etoposide or 5-FU for 24 hours. Untreated cells were used as control. Cell lysates were then immunoprecipitated with an antibody probe specific for caveolin-1 and immunoprecipitates subjected to immunoblotting analysis with anti-Ku70, anti-Bax and anti-caveolin-1 IgGs. (**B**) HCT116 colon cancer cells were transfected with HA-tagged wild type Ku70. One day after transfection, cells were treated with 5-FU for 24 hours. Untreated cells were used as control. Cells were then subjected to immunofluorescence analysis using antibody probes specific for the HA tag (green) and caveolin-1 (red). Nuclei were detected by DAPI staining (Blue). Yellow staining in the merged images shows co-localization between caveolin-1 and Ku70 only after 5-FU treatment (see arrows). Representative images are shown.

Once we demonstrated that Ku70 was a novel caveolin-1-interacting protein, we then asked whether binding of caveolin-1 to Ku70 was the molecular mechanism underlying the anti-apoptotic properties of caveolin-1. HCT116 cells were treated with either 5-FU or etoposide and the interaction between caveolin-1 and Ku70, before and after treatment with chemotherapeutic drugs, was evaluated by coimmunoprecipitation studies. We show in Figure 4A that the interaction between caveolin-1 and Ku70 was marginal under resting conditions but dramatically increased upon treatment with either 5-FU or etoposide. Consistent with these data, Ku70 was mainly localized in the nucleus and, to a lesser extent, diffuse in the cytoplasm (see arrowheads in Figure 4B) in HCT116 cells under resting conditions, away from caveolin-1, which was enriched at the plasma membrane (Figure 4B). In contrast, although nuclear localization was still evident, Ku70 was found to partially co-localize with caveolin-1 at the plasma membrane and intracellular caveolar membranes after 5-FU treatment (see arrows in Figure 4B). Interestingly, 5-FU, but not etoposide, also stimulated total caveolin-1 protein expression in HCT116 cells (Figure 4A, lower panel). This is consistent with the increased caveolin-1 mRNA level induced by 5-FU, but not etoposide, in HCT116 cells (Figure S5A), suggesting that 5-FU also promotes caveolin-1 protein expression through a transcriptional-dependent mechanism.

**Figure 5:**
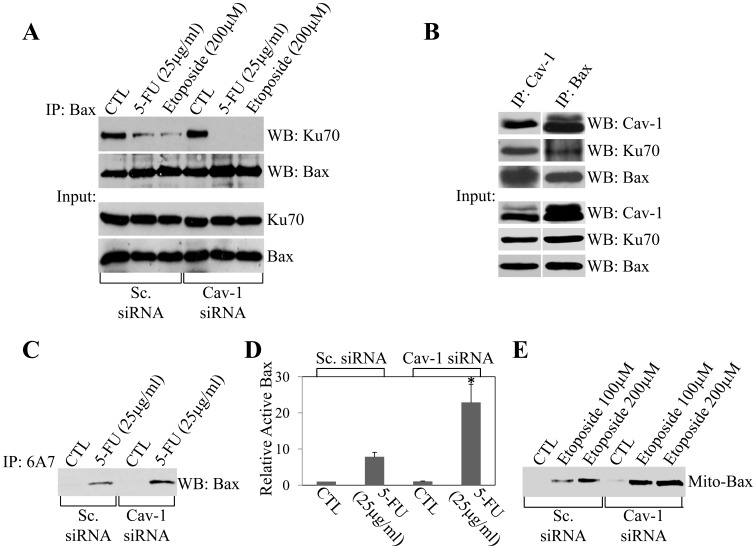
Knockdown of caveolin-1 protein expression enhances the dissociation of Bax from Ku70 and the activation of Bax induced by chemotherapeutic drugs. (A, C, D and E) HCT116 colon cancer cells were transfected with siRNA directed against caveolin-1. Transfection with scrambled siRNA was used as control. One day after transfection, HCT116 cells were treated with either 5-FU (A, C and D) or etoposide (A and E) for 24 hours. In (B), untransfected cells were treated with 5-FU for 24 hours. In (A), cell lysates were immunoprecipitated with an antibody probe specific for Bax and immunoprecipitates subjected to Western blotting analysis with anti-Ku70 and anti-Bax IgGs. In (B), cell lysates were immunoprecipitated with either anti-caveolin-1 IgGs or anti-Bax IgGs. Immunoprecipitates were then subjected to immunoblotting analysis with antibody probes specific for caveolin-1, Ku70 and Bax. In (C and D), cell lysates were immunoprecipitated with a Bax (6A7) antibody and immunoprecipitates subjected to immunoblotting analysis with anti-Bax IgGs. A blot that is representative of three independent experiments is shown in (C), quantification of Bax levels pulled down by the Bax (6A7) antibody is shown in (D). Values in (D) represent mean ± SEM; *P<0.001. In (E), mitochondrial fractions were isolated and expression of Bax in mitochondria was detected by immunoblotting analysis using an antibody probe specific for Bax.

Why does the chemotherapeutic drug-induced interaction between caveolin-1 and Ku70 act in an anti-apoptotic manner? Ku70 has been shown to bind to Bax under resting conditions. Release of Bax from Ku70 and the translocation of Bax to mitochondria are key steps in the signaling events that link an apoptotic stimulus to cell death. The molecular mechanisms that regulate these processes remain to be fully elucidated. Our data show that caveolin-1 acts as an anti-apoptotic protein (Figures 1 and 2) and chemotherapeutic drugs, which induce apoptosis, promote the interaction between caveolin-1 and Ku70 (Figures 4A and 4B). To investigate the functional significance of the binding of caveolin-1 to Ku70, we tested the hypothesis that binding of caveolin-1 to Ku70 inhibits apoptosis by limiting the release of Bax from Ku70. HCT116 cells were transfected with either scrambled or caveolin-1 siRNA and treated with either 5-FU or etoposide. Protein cell lysates were then immunoprecipitated with an antibody probe specific for Bax and immunoprecipitates subjected to immunoblotting analysis with anti-Ku70 IgGs. Figure 5A shows that both 5-FU and etoposide stimulated the release of Bax from Ku70 in scrambled siRNA-transfected cells. Importantly, the dissociation of Bax from Ku70 was potentiated in cells lacking caveolin-1. Thus, the interaction of caveolin-1 with Ku70 limits the release of Bax from Ku70 induced by chemotherapeutic drugs. In support of the existence of a caveolin-1/Ku70/Bax complex after treatment with chemotherapeutic drugs, we show in Figure 4A that both 5-FU and etoposide promoted the interaction between caveolin-1 and Bax, and in Figure 5B that caveolin-1, Ku70 and Bax are all found by immunoblotting analysis after immunoprecipitation of protein cell lysates from 5-FU-treated HCT116 cells with antibody probes specific for either caveolin-1 or Bax.

### Knockdown of Caveolin-1 Protein Expression Potentiates the Chemotherapeutic Drug-induced Conformational Change of Bax

Activation of Bax is associated with a conformational change that leads to exposure of the N terminus and can be detected using the anti-Bax antibody clone 6A7 [29], [30]. We show in Figures 5C and 5D that 6A7 antibody immunoprecipitated Bax only in HCT116 cells treated with the apoptotic stimulus 5-FU but not in control cells. Knockdown of caveolin-1 protein expression by siRNA significantly potentiated binding of the 6A7 antibody to Bax in cells treated with 5-FU (Figures 5C and 5D). However, knockdown of caveolin-1 protein expression by itself in untreated cells failed to promote the conformational change of Bax (Figures 5C and 5D). Since our results show that chemotherapeutic drugs stimulate the binding of caveolin-1 with Ku70 and the interaction of Bax with Ku70 is known to prevent the activation of Bax, our data suggest that caveolin-1 forms a complex with Ku70 that inhibits the activation of Bax. The dissociation of caveolin-1 from Ku70 is not sufficient to activate Bax but potentiates Bax activation induced by chemotherapeutic drugs. Consistent with these results, chemotherapeutic drug-induced translocation of Bax to mitochondria was enhanced in HCT116 cells in which caveolin-1 protein expression was reduced by siRNA, as compared to HCT116 cells expressing scrambled siRNA (Figure 5E). We conclude that binding of caveolin-1 to Ku70 limits the release of Bax from Ku70 and the activation of Bax induced by chemotherapeutic drugs.

### Enhancement of Apoptosis Induced by the Knockdown of Caveolin-1 Protein Expression is Dependent upon the Expression of Bax

**Figure 6:**
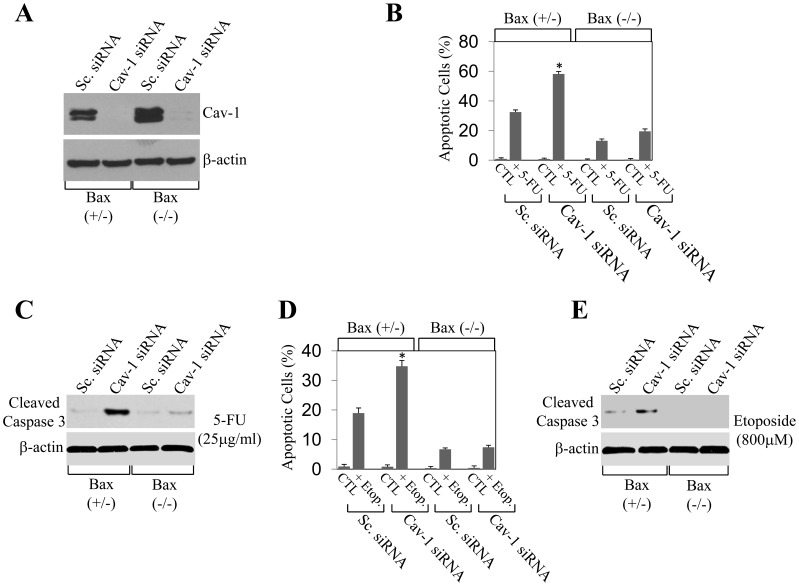
Knockdown of caveolin-1 protein expression fails to enhance apoptosis induced by chemotherapeutic drugs in the absence of Bax expression. HCT116 and HCT116 Bax null cells were transfected with siRNA directed against caveolin-1. Transfection with scrambled siRNA was used as control. In (A), expression of endogenous caveolin-1 was detected by immunoblotting analysis 48 hours after transfection using an antibody probe specific for caveolin-1. Immunoblotting with anti-β-actin IgGs was done to show equal loading. In (B–E), 24 hours after transfection, cells were treated with either 5-FU (25 µg/ml) for 48 h (B and C) or etoposide (Etop.; 800 µM) for 24 h (D and E). Untreated cells were used as control. In (B and D), cells were stained with DAPI and the number of cells showing nuclear condensation was quantified. Values represent mean ± SEM; *P<0.001. In (C and E), cells were subjected to immunoblotting analysis with antibody probes specific for cleaved caspase 3. Immunoblotting with anti-β-actin IgGs was done to show equal loading.

If knockdown of caveolin-1 protein expression potentiates apoptosis in colon cancer cells by enhancing the release of Bax from Ku70, one would not expect potentiation of apoptosis by knockdown of caveolin-1 in HCT116 cells lacking Bax expression. To directly test this possibility, we took advantage of HCT116 Bax (−/−) null isogenic cells [31], which do not express Bax (data not shown). Caveolin-1 protein expression was knockdown by siRNA in HCT116 and HCT116 Bax null cells (Figure 6A). Cells were then treated with either 5-FU or etoposide and apoptosis was quantified by DAPI staining. We demonstrate that knockdown of caveolin-1 protein expression by siRNA potentiated 5-FU-induced apoptosis in HCT116 cells by 81% but only by 46% in HCT116 Bax null cells (Figure 6B). Similarly, knockdown of caveolin-1 protein expression by siRNA enhanced etoposide-induced apoptosis in HCT116 cells by 84% but only by 10% in HCT116 Bax null cells (Figure 6D). Consistent with these data, the enhanced activation of caspase 3 by downregulation of caveolin-1 expression following treatment with either 5-FU or etoposide observed in HCT116 cells was dramatically inhibited in HCT116 Bax null cells (Figures 6C and 6E, respectively). Thus, Bax expression plays a central role in the potentiation of chemotherapeutic drug-induced apoptosis following knockdown of caveolin-1 in HCT116 cells. The fact that we observed apoptosis after 5-FU treatment even in HCT116 Bax null cells transfected with scrambled siRNA (Figures 6B and 6D) suggests that apoptosis in these cells can also be Bax-independent, confirming previously reported data showing Bax-independent apoptosis in these cells [32], [33]. Finally, the 46% increase in apoptosis induced by 5-FU found in caveolin-1 siRNA-expressing HCT116 Bax null cells, as compared to scrambled siRNA-expressing HCT116 Bax null cells (Figure 6B), suggests that, in addition to Bax-dependent apoptosis, caveolin-1 may also inhibit, to a certain extent, Bax-independent cell death following treatment with 5-FU.

### The Interaction of Ku70 with Caveolin-1 is Required for the Ku70-dependent Protection against Chemotherapeutic Drug-induced Apoptosis

**Figure 7:**
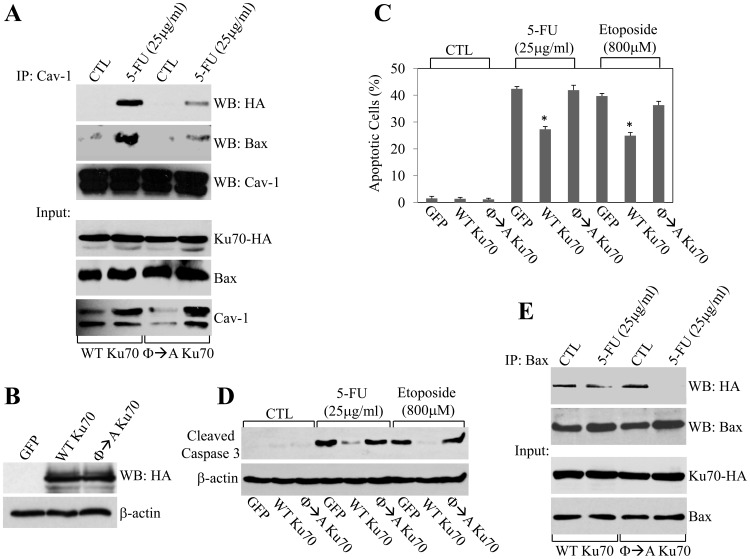
Φ→A Ku70 fails to interact with caveolin-1, to protect against chemotherapeutic drug-induced apoptosis and to limit the 5-FU-induced dissociation of Bax from Ku70. HCT116 cells were transfected with either wild type Ku70-HA (WT Ku70) or Φ→A Ku70-HA (Φ→A Ku70) and treated with different concentrations of either 5-FU (for 24 h in A and E; for 48 h in C and D) or etoposide (for 24 h in C and D). Untreated cells were used as control. In (A), cell lysates were immunoprecipitated with an antibody probe specific for caveolin-1 and immunoprecipitates subjected to immunoblotting analysis with anti-HA, anti-Bax and anti-caveolin-1 IgGs. In (B), total expression of WT Ku70-HA and Φ→A Ku70-HA was detected by immunoblotting analysis with anti-HA IgGs before drug treatment. In (C), cells were stained with DAPI and the number of cells showing nuclear condensation was quantified. Values represent mean ± SEM; *P<0.001. In (D), cells were subjected to immunoblotting analysis with antibody probes specific for cleaved caspase 3. Immunoblotting with anti-β-actin IgGs was done to show equal loading. In (E), cell lysates were immunoprecipitated with and antibody probe specific for Bax and immunoprecipitates subjected to Western blotting analysis with anti-HA and anti-Bax IgGs.

Overexpression of Ku70 has been shown to protect against apoptosis [22], [25]. Based on our data showing that binding of caveolin-1 to Ku70 protects the cells against apoptosis induced by chemotherapeutic drugs, we would predict less protection against apoptosis by a mutant form of Ku70 that cannot bind to caveolin-1. Since we show in Figure 3 that caveolin-1 interacts with Ku70, to directly test our hypothesis, we generated a mutant form of Ku70 in which the critical aromatic residues of the caveolin binding domain of Ku70 were mutated to alanines (Φ→A-Ku70). We show that the *in vitro* binding of Φ→A-Ku70 to caveolin-1 was greatly inhibited, as compared to wild type Ku70 (Figures S5B and S5C). We then overexpressed wild type Ku70 and Φ→A-Ku70 in HCT116 cells. Cells were treated with 5-FU and the interaction of caveolin-1 with Ku70 and Bax was examined by coimmunoprecipitation studies. We find that 5-FU promoted the interaction of caveolin-1 with wild type Ku70 and Bax in cells overexpressing wild type Ku70 (Figure 7A). In contrast, the interaction of caveolin-1 with Φ→A-Ku70 and Bax was only marginally stimulated by 5-FU treatment in cells overexpressing Φ→A-Ku70 (Figure 7A). Consistent with our findings, Φ→A-Ku70, similarly to wild type Ku70 (Figure 4B), did not co-localize with caveolin-1 in HCT116 cells under resting conditions, being mainly localized in the nucleus and, to a lesser extent, diffuse in the cytoplasm (see arrowheads in Figure S6). However, in contrast to wild type Ku70 (Figure 4B), 5-FU failed to promote the co-localization of Φ→A-Ku70 with caveolin-1 in HCT116 cells (Figure S6). Interestingly, overexpression of wild type Ku70, but not Φ→A-Ku70, promoted a ∼40% decrease in apoptosis induced by either 5-FU or etoposide (Figures 7B and 7C). In support to this data, we show in Figure 7D that wild type Ku70, in contrast to Φ→A-Ku70, prevented the activation of caspase 3 by either 5-FU or etoposide and that overexpression of wild type Ku70, but not Φ→A-Ku70, limited the Ku70/Bax dissociation promoted by 5-FU in HCT116 cells (Figure 7E). These results support our conclusion that the interaction of Ku70 with caveolin-1 limits the chemotherapeutic drug-induced release of Bax from Ku70 and therefore Bax-dependent apoptosis.

## Discussion

Apoptosis is the process of programmed cell death adopted by multicellular organisms for proper development and maintenance of adult individuals. Defective apoptosis has been linked to pathological conditions. Excessive apoptosis may lead to atrophy whereas reduced apoptosis may lead to uncontrolled cell proliferation and cancer. Apoptosis is also a major cytotoxic effect of chemotherapy. Thus, understanding, at the molecular level, how apoptosis is regulated will not only bring insight into a fundamental biological event but also contribute to the development of more efficient therapeutic interventions for the treatment of cancer. 5-fluorouracil is a chemotherapeutic drug that has been the cornerstone of treatment for patients with colorectal cancer for almost four decades. 5-FU blocks the enzyme thymidylate synthase and inhibits both RNA and DNA synthesis. In sensitive cells, 5-FU induces apoptosis. Unfortunately, tumor responses to 5-FU is limited by toxicity to tissues containing highly proliferative cell populations [34]. In this report, we show that knockdown of caveolin-1 protein expression in colon cancer cells enhances apoptosis induced by 5-FU and etoposide. Mechanistically, our data reveal a novel anti-apoptotic mechanism in which the binding of caveolin-1 to Ku70 limits the dissociation of Bax from Ku70 and the activation of Bax induced by apoptotic stimuli. Consistent with this conclusion, we show that the enhancement of 5-FU-induced apoptosis after knockdown of caveolin-1 protein expression is dramatically prevented in the absence of Bax expression. Therefore, caveolin-1 acts as an anti-apoptotic molecule in colon cancer cells by inhibiting Bax-dependent cell death.

Overexpression of caveolin-1 was linked to drug-resistance of cancer cells, including colon cancer cells [35], [36]. Our findings contribute to explain, at the molecular level, the role of caveolin-1 in drug resistance of cancer cells. Increased levels of caveolin-1 may promote resistance to chemotherapeutic drugs by bolstering the inhibitory action of caveolin-1 on the Ku70-Bax complex. In fact, in the presence of elevated caveolin-1 levels, the release of Bax from Ku70 induced by chemotherapeutic drugs would be further inhibited. This mechanism would also be consistent with a proposed scenario in which caveolin-1 expression is lost in the initial phases of cancer development but returns to high levels at later stages when the tumor has acquired multi-drug resistance properties (reviewed in [37]).

Caveolin-1 was recently shown to modulate the cellular response to DNA damage by regulating both homologous recombination (HR) and non-homologous end joining (NHEJ) repair pathways [38]. Interestingly, Ku70 was originally discovered as a molecule involved in the repair of DNA double-strand breaks by non-homologous end joining (NHEJ). Since we demonstrate in this report that Ku70 is a novel caveolin-1 binding protein, our findings suggest that caveolin-1 may regulate DNA repair through modulation of Ku70 functions which are independent of apoptosis. How caveolin-1 regulates Ku70 in the context of DNA repair mechanisms is currently under investigation.

Overexpression of Ku70 has been shown to protect against apoptosis [22], [25]. Our data contribute to explain this previous finding at the molecular level. We confirmed that overexpression of wild type Ku70 inhibits both 5-FU- and etoposide-induced apoptosis of colon cancer cells. More importantly, we show that a mutant form of Ku70 that cannot interact with caveolin-1 (Φ→A Ku70) fails to inhibit chemotherapeutic drug-induced apoptosis. Thus, the interaction of Ku70 with caveolin-1 is critical for the ability of Ku70 to inhibit apotosis. We also found that overexpression of Φ→A Ku70, in contrast to wild type Ku70, fails to inhibit the Bax/Ku70 dissociation after stimulation with chemotherapeutic drugs, which explains, at the molecular level, why Φ→A Ku70 fails to protect against chemotherapeutic drug-induced apoptosis. Thus, under cellular conditions where Ku70 fails to interact with caveolin-1 (i.e. cells in which caveolin-1 protein expression is knockdown by siRNA or cells expressing the Φ→A mutant form of Ku70 that does not interact with caveolin-1), the ability of Ku70 to limit the release of Bax is prevented. Together, our data indicate that disruption of the interaction between caveolin-1 and Ku70 enhances apoptosis and propose the caveolin-1/Ku70 complex as a novel potential therapeutic target for the treatment of colon cancer.

## Supporting Information

Figure S1 **Quantification of nuclear condensation after knockdown of caveolin-1 protein expression in 5-FU-treated HCT116 colon cancer cells.** HCT116 colon cancer cells were transfected with siRNA directed against caveolin-1. Transfection with scrambled siRNA was used as control. One day after transfection, cells were treated with 5-FU for different periods of time. Untreated cells were used as control. Cells were then stained with DAPI. Representative images are shown in (A). Arrows show examples of cells with nuclear condensation. The number of cells showing nuclear condensation was quantified in (B). Values represent mean ± SEM; *P<0.001.
(TIF)

Figure S2 **Knockdown of caveolin-1 protein expression reduces the number of cells in the S phase of the cell cycle.** HCT116 colon cancer cells were transfected with siRNA directed against caveolin-1. Transfection with scrambled siRNA was used as control. One day after transfection, HCT116 cells were collected, incubated with propidium iodide and subjected to FACS analysis with the use of a fluorescence-activated cell sorter (FACStar plus; Becton Dickinson). A representative cell cycle analysis experiment is shown. Values represent mean from independent experiments.
(TIF)

Figure S3 **Quantification of nuclear condensation after knockdown of caveolin-1 protein expression in 5-FU-treated HT29 colon cancer cells.** HT29 colon cancer cells were transfected with siRNA directed against caveolin-1. Transfection with scrambled siRNA was used as control. One day after transfection, cells were treated with 5-FU for different periods of time. Untreated cells were used as control. Cells were then stained with DAPI. Representative images are shown in (**A**). Arrows show examples of cells with nuclear condensation. The number of cells showing nuclear condensation was quantified in (**B**). Values represent mean ± SEM; *P<0.001.
(TIF)

Figure S4 **Knockdown of caveolin-1 protein expression inhibits long-term survival of HCT116 cells after treatment with etoposide.** HCT116 colon cancer cells were transfected with siRNA directed against caveolin-1. Transfection with scrambled siRNA was used as control. One day after transfection, HCT116 cells were treated with different concentrations of etoposide for 1 hour. Untreated cells were used as control. Cells were then cultured for 7 days and stained with crystal violet. A representative crystal violet staining after etoposide treatment is shown in (**A**). Quantification of crystal violet staining after etoposide treatment is shown in (**B**). Values represent mean ± SEM; *P<0.001; ^#^ *P*<0.005.
(TIF)

Figure S5 **5-FU, but not etoposide, increases caveolin-1 mRNA levels in HCT116 cells. The binding of Φ →A Ku70 to caveolin-1 is dramatically compromised.** (**A**) HCT116 cells were treated with either 5-FU or etoposide for 24 hours. Untreated cells were used as control. RNA was extracted and RT-PCR was performed using primers specific for human caveolin-1. Amplification of GAPDH was performed by RT-PCR as an internal control. A representative blot is shown. (**B**) Ponceau S staining of GST alone and the GST-caveolin-1 (82–101) fusion protein. (**C**) GST-caveolin-1 fusion protein pull-down assays were performed using cell lysates from HCT116 cells transiently transfected with either wild type Ku70-HA or Φ→A Ku70-HA. A blot that is representative of two independent experiments is shown.
(TIF)

Figure S6 Φ**→A Ku70 fails to co-localize with caveolin-1 after treatment with 5-FU.** HCT116 colon cancer cells were transfected with HA-tagged Φ→A Ku70. One day after transfection, cells were treated with 5-FU for 24 hours. Untreated cells were used as control. Cells were then subjected to immunofluorescence analysis using antibody probes specific for the HA tag (green) and caveolin-1 (red). Nuclei were detected by DAPI staining (Blue). Representative images are shown.
(TIF)
