# Correction: Interaction of Caveolin-1 with Ku70 Inhibits Bax-Mediated Apoptosis

**DOI:** 10.1371/annotation/83804d5a-9c9b-4b9a-9aa3-cb6da8a78fe9

**Published:** 2012-09-19

**Authors:** Huafei Zou, Daniela Volonte, Ferruccio Galbiati

There were errors in the Funding section. The correct funding information is as follows: This work was supported by grants from the National Institute on Aging (R01-AG030636) (to F.G.), the American Heart Association (12SDG8800012) and the Competitive Medical Research Fund of the UPMC Health System (to D.V.) The funders had no role in study design, data collection and analysis, decision to publish, or preparation of the manuscript. 

