# Supplementary figures and images for: Interaction of Caveolin-1 with Ku70 Inhibits Bax-Mediated Apoptosis
Source: PLoS One. 2012 Jun 20;7(6):e39379. doi: 10.1371/journal.pone.0039379 (PMC3380016; doi:10.1371/journal.pone.0039379)

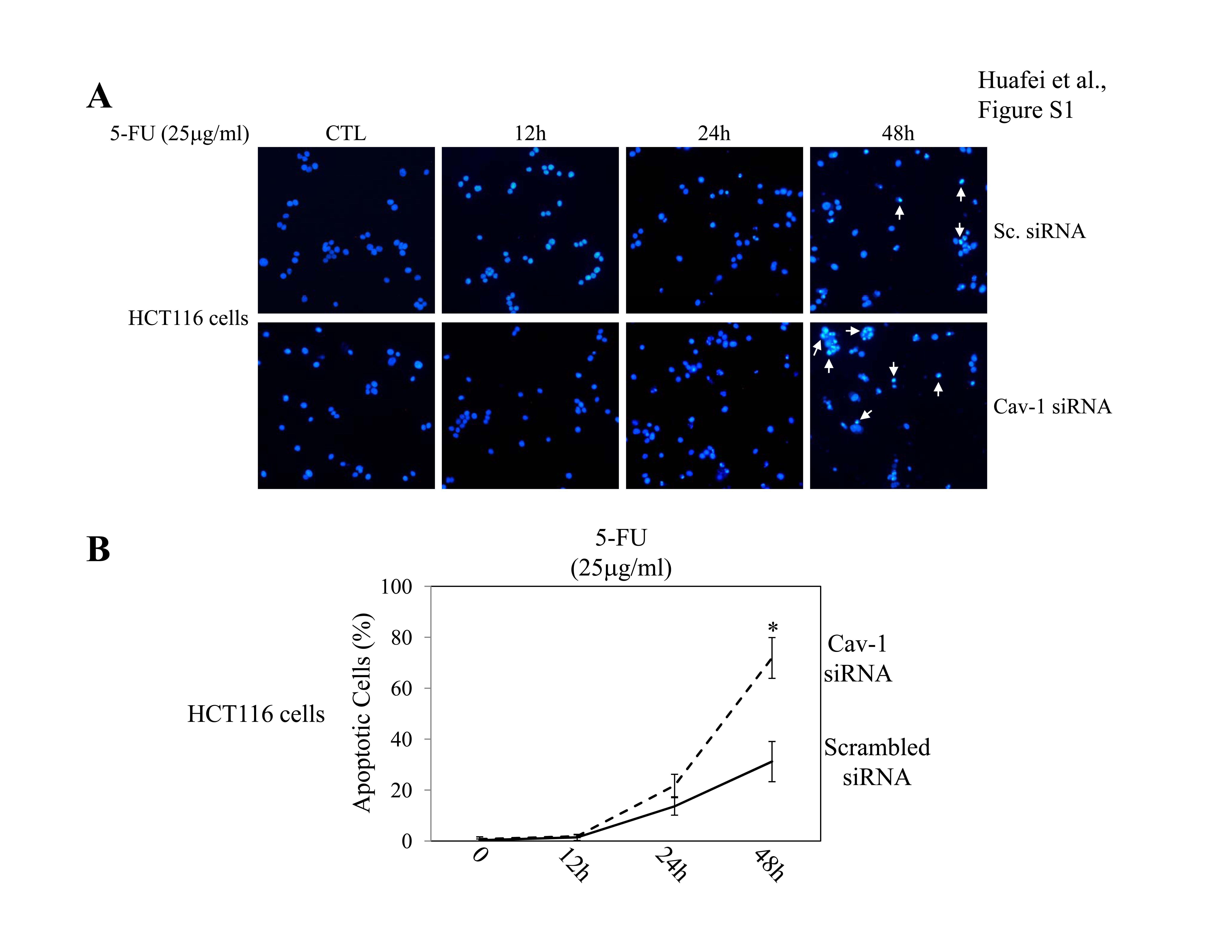

Supplement: Figure S1 — Quantification of nuclear condensation after knockdown of caveolin-1 protein expression in 5-FU-treated HCT116 colon cancer cells. HCT116 colon cancer cells were transfected with siRNA directed against caveolin-1. Transfection with scrambled siRNA was used as control. One day after transfection, cells were treated with 5-FU for different periods of time. Untreated cells were used as control. Cells were then stained with DAPI. Representative images are shown in (A). Arrows show examples of cells with nuclear condensation. The number of cells showing nuclear condensation was quantified in (B). Values represent mean ± SEM; *P<0.001. (TIF) [file pone.0039379.s001.tif]

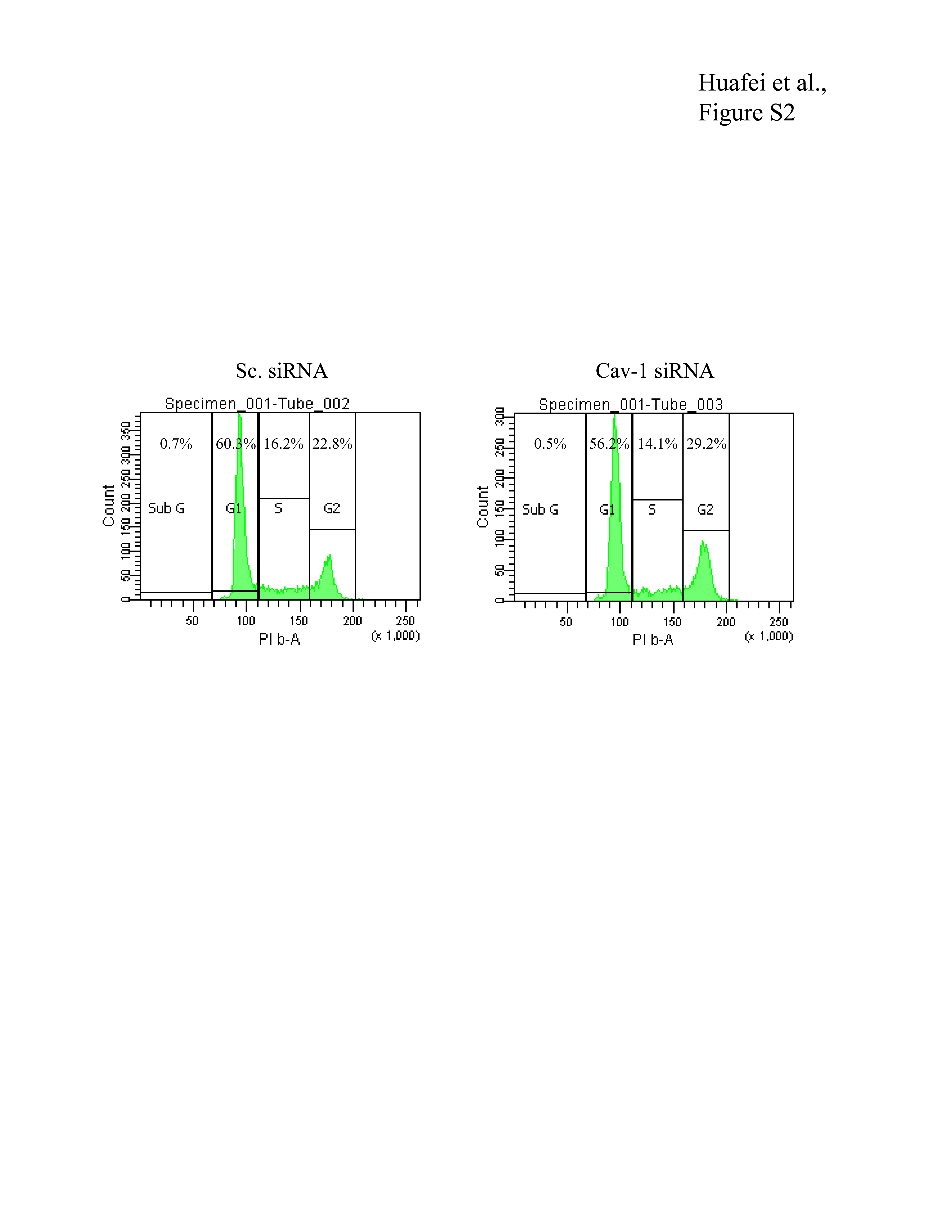

Supplement: Figure S2 — Knockdown of caveolin-1 protein expression reduces the number of cells in the S phase of the cell cycle. HCT116 colon cancer cells were transfected with siRNA directed against caveolin-1. Transfection with scrambled siRNA was used as control. One day after transfection, HCT116 cells were collected, incubated with propidium iodide and subjected to FACS analysis with the use of a fluorescence-activated cell sorter (FACStar plus; Becton Dickinson). A representative cell cycle analysis experiment is shown. Values represent mean from independent experiments. (TIF) [file pone.0039379.s002.tif]

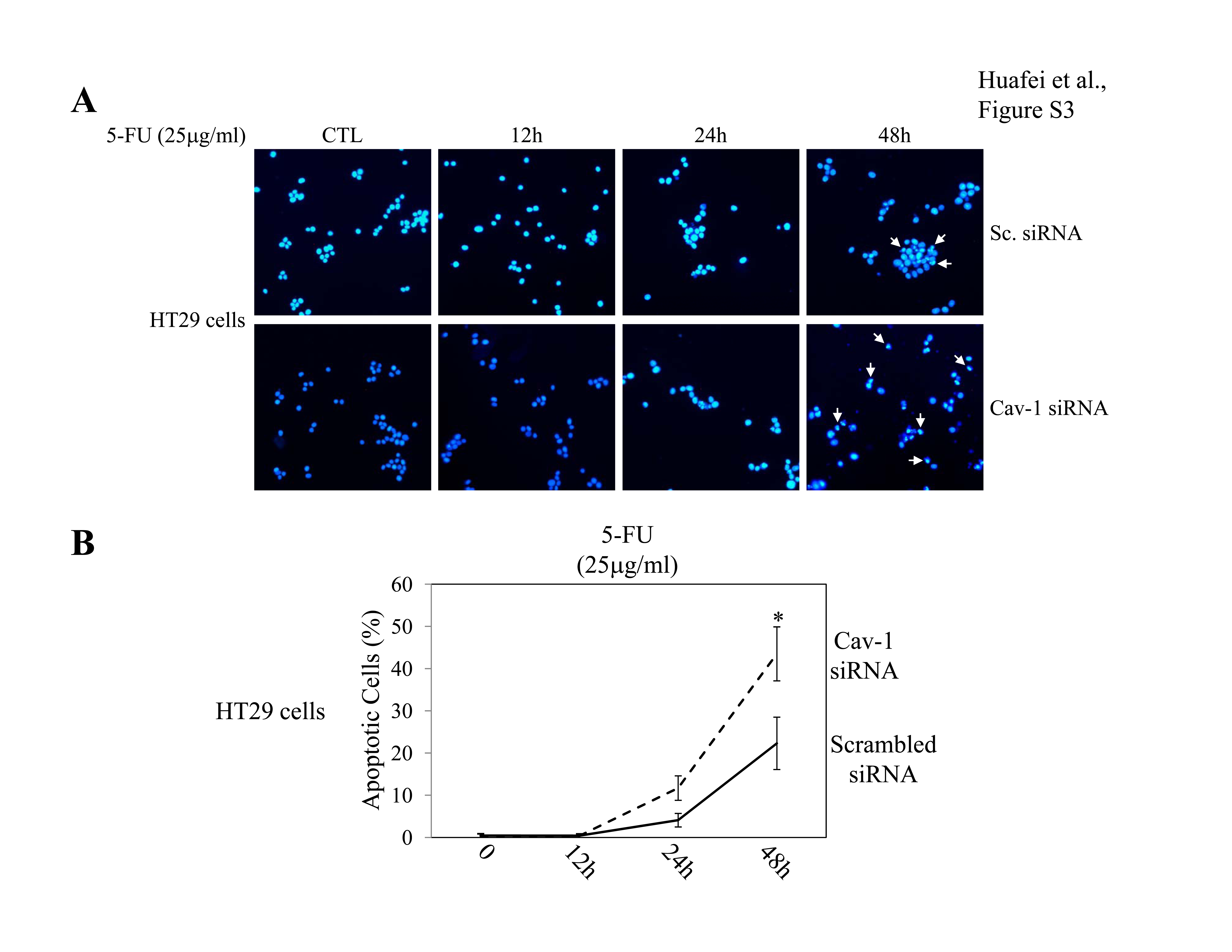

Supplement: Figure S3 — Quantification of nuclear condensation after knockdown of caveolin-1 protein expression in 5-FU-treated HT29 colon cancer cells. HT29 colon cancer cells were transfected with siRNA directed against caveolin-1. Transfection with scrambled siRNA was used as control. One day after transfection, cells were treated with 5-FU for different periods of time. Untreated cells were used as control. Cells were then stained with DAPI. Representative images are shown in (A). Arrows show examples of cells with nuclear condensation. The number of cells showing nuclear condensation was quantified in (B). Values represent mean ± SEM; *P<0.001. (TIF) [file pone.0039379.s003.tif]

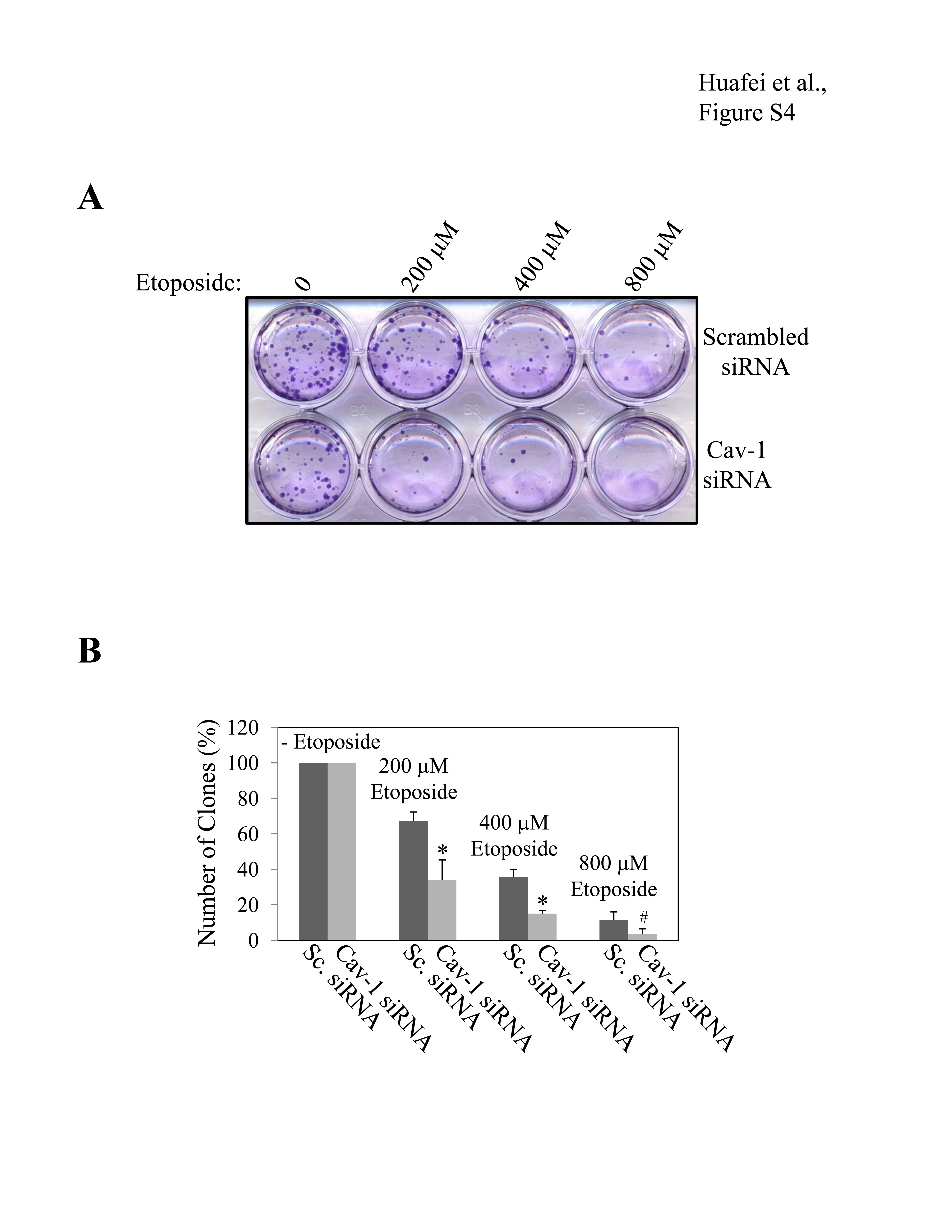

Supplement: Figure S4 — Knockdown of caveolin-1 protein expression inhibits long-term survival of HCT116 cells after treatment with etoposide. HCT116 colon cancer cells were transfected with siRNA directed against caveolin-1. Transfection with scrambled siRNA was used as control. One day after transfection, HCT116 cells were treated with different concentrations of etoposide for 1 hour. Untreated cells were used as control. Cells were then cultured for 7 days and stained with crystal violet. A representative crystal violet staining after etoposide treatment is shown in (A). Quantification of crystal violet staining after etoposide treatment is shown in (B). Values represent mean ± SEM; *P<0.001; # P<0.005. (TIF) [file pone.0039379.s004.tif]

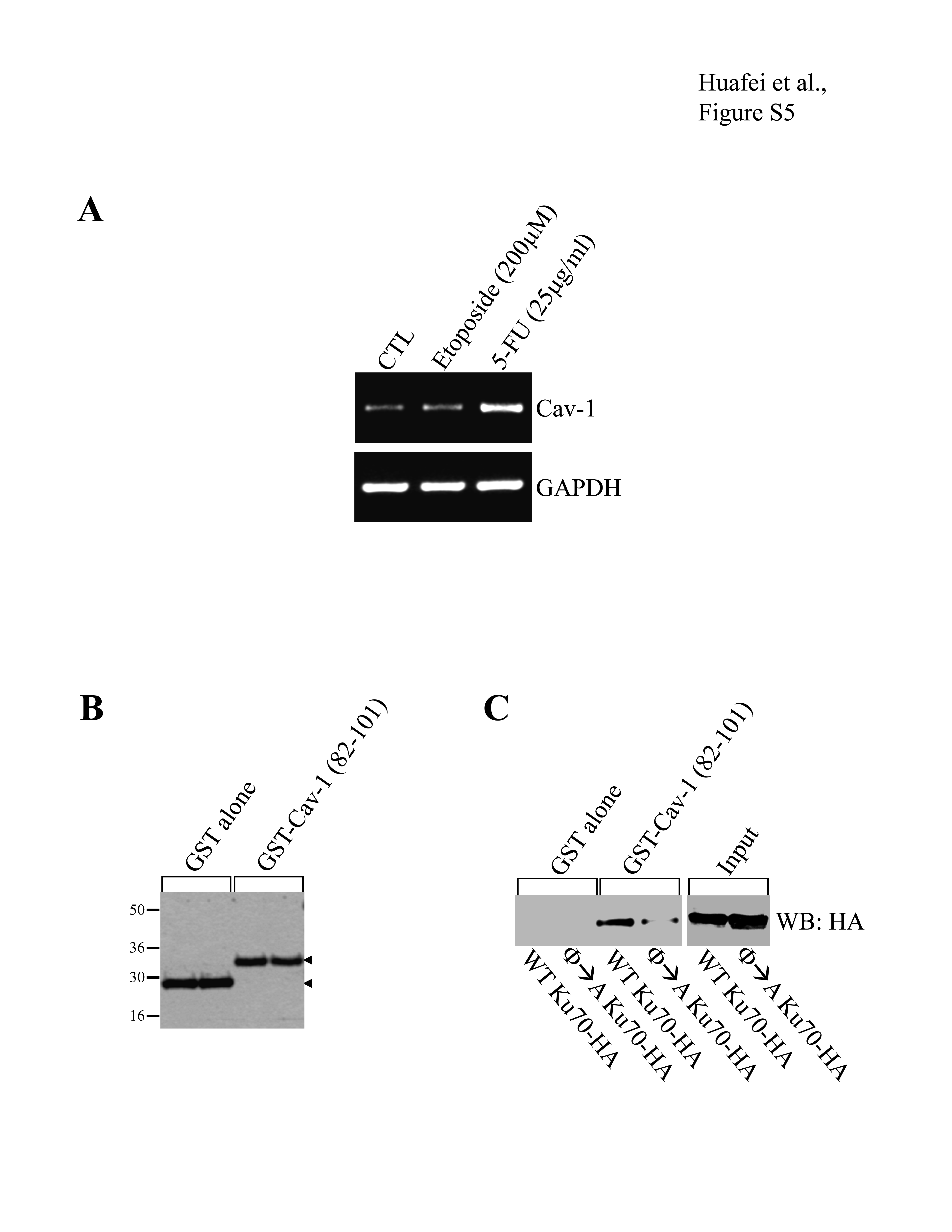

Supplement: Figure S5 — 5-FU, but not etoposide, increases caveolin-1 mRNA levels in HCT116 cells. The binding of Φ →A Ku70 to caveolin-1 is dramatically compromised. (A) HCT116 cells were treated with either 5-FU or etoposide for 24 hours. Untreated cells were used as control. RNA was extracted and RT-PCR was performed using primers specific for human caveolin-1. Amplification of GAPDH was performed by RT-PCR as an internal control. A representative blot is shown. (B) Ponceau S staining of GST alone and the GST-caveolin-1 (82–101) fusion protein. (C) GST-caveolin-1 fusion protein pull-down assays were performed using cell lysates from HCT116 cells transiently transfected with either wild type Ku70-HA or Φ→A Ku70-HA. A blot that is representative of two independent experiments is shown. (TIF) [file pone.0039379.s005.tif]

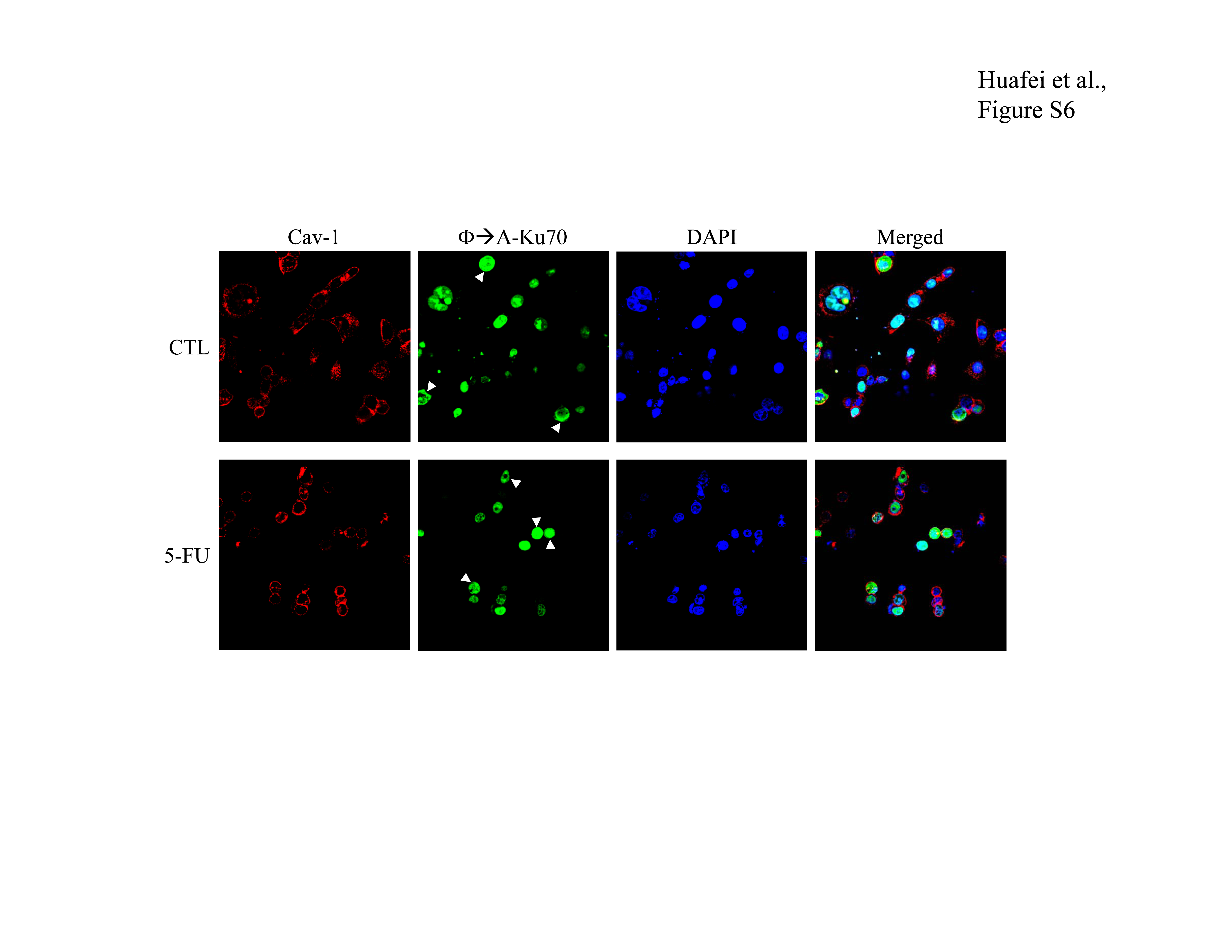

Supplement: Figure S6 — Φ→A Ku70 fails to co-localize with caveolin-1 after treatment with 5-FU. HCT116 colon cancer cells were transfected with HA-tagged Φ→A Ku70. One day after transfection, cells were treated with 5-FU for 24 hours. Untreated cells were used as control. Cells were then subjected to immunofluorescence analysis using antibody probes specific for the HA tag (green) and caveolin-1 (red). Nuclei were detected by DAPI staining (Blue). Representative images are shown. (TIF) [file pone.0039379.s006.tif]
